# Phytochemical Profiling and Proteomic Insights into the Anti-Inflammatory Effects of Jing Guan Fang in LPS-Stimulated Macrophages

**DOI:** 10.3390/antiox15081010

**Published:** 2026-08-13

**Authors:** Seungyeon Yeon, Muhammad A. Alsherbiny, Yu-Ting Sun, Mitchell N. Low, Chun Guang Li

**Affiliations:** 1 NICM Health Research Institute, Western Sydney University, Westmead, NSW 2145, Australia; maz.ali@westernsydney.edu.au (M.A.A.); mitchell.low@westernsydney.edu.au (M.N.L.); 2 Traditional Chinese Medicine, School of Health Sciences, Western Sydney University, Campbelltown, NSW 2560, Australia; yu-ting.sun@westernsydney.edu.au; 3 ARC Training Centre for Facilitated Advancement of Australia’s Bioactives (FAAB), Macquarie University, North Ryde, NSW 2109, Australia

**Keywords:** Jing Guan Fang, herbal medicine, natural products, phytochemical profiling, proteomics, secretome, macrophages, inflammation, cytokines

## Abstract

Jing Guan Fang (JGF) is a traditional multi-herb formula used for hyperinflammatory conditions associated with severe viral infections; however, its protein-level effects in macrophage-mediated inflammation remain incompletely characterised. In this study, we combined ultra-performance liquid chromatography–quadrupole time-of-flight mass spectrometry (UPLC-QTOF-MS)-based phytochemical profiling, functional anti-inflammatory assays, and discovery-level proteomic and secretome analyses to investigate the anti-inflammatory effects of JGF in lipopolysaccharide (LPS)-stimulated RAW 264.7 macrophages. Phytochemical profiling revealed predominantly flavonoid- and phenolic-related annotated features, together with selected terpenoid- and alkaloid-related annotations. Functionally, JGF treatment was associated with reduced nitric oxide production and differential effects on pro-inflammatory cytokine release, with a clearer concentration-dependent reduction in interleukin-6 (IL-6) and a more variable tumour necrosis factor-α (TNF-α) response, without reducing cell viability. Label-free quantitative proteomics suggested that JGF treatment was associated with modulation of LPS-induced protein changes, including reduced abundance of inflammation-associated proteins such as myristoylated alanine-rich C-kinase substrate (MARCKS) and inducible nitric oxide synthase (NOS2), together with partial restoration of selected regulatory proteins such as AIMP1 and AIMP2. Secretome analysis further suggested that JGF reduced extracellular inflammatory and tissue-remodelling-related mediators, including SERPINE1/PAI-1. Exploratory pathway analysis indicated that JGF treatment was associated with changes in inflammation-related and oxidative stress-related signalling networks. Collectively, these findings provide discovery-level molecular insights into the anti-inflammatory effects of JGF in LPS-stimulated macrophages and support further targeted validation of its pharmacological activity as a candidate multi-component anti-inflammatory formula.

## 1. Introduction

Dysregulated inflammation is a central feature of severe respiratory viral infections and hyperinflammatory responses, including cytokine storm-associated conditions [1,2,3,4]. Excessive macrophage activation contributes to the production of inflammatory mediators such as nitric oxide (NO), interleukin-6 (IL-6), and tumour necrosis factor-alpha (TNF-α), which can amplify local and systemic inflammatory responses [2,3,4]. These challenges have renewed interest in multi-component natural products and traditional herbal formulas that may modulate inflammatory networks through complementary biological activities [5].

Traditional herbal formulas represent a valuable source of bioactive compounds with potential anti-inflammatory and immunomodulatory activity. Unlike single-compound interventions, multi-herb formulas contain diverse phytochemical classes that may collectively influence signalling pathways involved in inflammation, oxidative stress, and immune regulation. However, their chemical complexity creates challenges for interpretation, particularly when phytochemical composition and biological activity are investigated separately. Combining phytochemical profiling with discovery-level proteomic analysis may help provide molecular insight into how complex herbal preparations are associated with cellular inflammatory responses.

Jing Guan Fang (JGF) is a traditional five-herb formula used in Taiwan in the context of respiratory viral infection-related inflammatory conditions [6,7]. JGF consists of *Forsythia suspensa* (Thunb.) Vahl, *Scutellaria baicalensis* Georgi, *Bupleurum chinense* DC., *Magnolia officinalis* Rehder & E.H. Wilson, and *Agastache rugosa* Kuntze. Previous studies have reported chemical characterisation, anti-SARS-CoV-2-related activity, and immunomodulatory effects of JGF, including attenuation of coronavirus envelope protein-induced proinflammatory responses, interference with host factors involved in SARS-CoV-2 entry, and modulation of macrophage inflammatory signalling pathways [6,7,8]. The botanical composition of JGF suggests the presence of diverse phytochemical classes, including flavonoids, phenolics, lignans, and selected terpenoid-related constituents, which may contribute to its reported anti-inflammatory activity. Nevertheless, the protein-level effects of the complete JGF formula in macrophage-mediated inflammation remain incompletely characterised.

Most previous investigations of traditional herbal formulas have relied heavily on clinical observation, targeted assays, or in silico network pharmacology approaches [9]. While these studies are useful for hypothesis generation, they do not fully capture the protein-level changes associated with complete herbal formulas in inflammatory cells. In particular, the relationship between the phytochemical profile of JGF and its effects on macrophage inflammatory signalling remains insufficiently characterised. Proteomics provides an unbiased approach for identifying treatment-associated protein networks and regulatory pathways altered by inflammatory stimulation [10], while ultra-performance liquid chromatography–quadrupole time-of-flight mass spectrometry (UPLC-QTOF-MS)-based phytochemical profiling enables chemical characterisation of complex herbal preparations [11]. Together, these approaches can provide discovery-level molecular insight into the anti-inflammatory effects of multi-component herbal formulas.

In this study, we combined UPLC-QTOF-MS-based phytochemical profiling, functional anti-inflammatory assays, and discovery-level proteomic and secretome analyses to investigate the anti-inflammatory effects of JGF in lipopolysaccharide (LPS)-stimulated RAW 264.7 macrophages. UPLC-QTOF-MS-based phytochemical profiling was used to characterise the chemical features of a capsule-derived JGF extract. The anti-inflammatory effects of JGF were assessed by measuring NO, IL-6, and TNF-α production together with cell viability. Finally, label-free quantitative proteomics was applied to examine JGF-associated changes in both the cellular proteome and secretome. This study aimed to provide complementary discovery-level insight into the phytochemical composition of JGF and its macrophage-mediated anti-inflammatory effects.

## 2. Materials and Methods

### 2.1. Preparation of Jing Guan Fang (JGF) Extract

Jing Guan Fang (JGF) capsules were obtained from Sun Ten Pharmaceutical Co., Ltd. (New Taipei City, Taiwan). The test material used in this study was the capsule-derived preparation designated as “JGF Capsule” in the retained experimental records. The capsule contents were extracted with absolute ethanol (ChemSupply Australia, Gillman, SA, Australia), after which the solvent was removed under reduced pressure, and the resulting residue was resuspended in ultrapure water and lyophilised. The lyophilised capsule-derived extract was stored at −80 °C until use.

For cellular assays, a 50 mg/mL stock solution was prepared by dissolving 100.0 mg of the lyophilised JGF extract in 2 mL of a 1:1 (*v*/*v*) mixture of phosphate-buffered saline (PBS) and dimethyl sulfoxide (DMSO). The stock solution was diluted in serum-free Dulbecco’s Modified Eagle’s Medium (DMEM; Lonza, Norwest, NSW, Australia) to obtain the required JGF treatment concentrations, with a maximum final treatment concentration of 150 µg/mL. A concentration of 100 µg/mL was used for the proteomic and secretome analyses.

### 2.2. UPLC-QTOF-MS-Based Phytochemical Profiling

The lyophilised JGF extract was dissolved in a solvent mixture of water, acetonitrile, and methanol (2:1:1, *v*/*v*/*v*) to a final concentration of 5 mg/mL and filtered through a 0.2 µm polytetrafluoroethylene (PTFE) syringe filter prior to analysis. UPLC-QTOF-MS data were acquired using an ACQUITY UPLC system coupled to a SYNAPT G2-Si quadrupole time-of-flight mass spectrometer (Waters Corporation, Milford, MA, USA). Instrument control and data acquisition were performed using MassLynx software (version 4.2; Waters Corporation, Milford, MA, USA). Chromatographic separation was performed on an ACQUITY UPLC HSS-T3 column (100 mm × 2.1 mm, 1.8 µm; Waters Corporation, Milford, MA, USA) maintained at 45 °C. The mobile phases consisted of water (A) and acetonitrile (B), both containing 0.1% (*v*/*v*) formic acid, with a flow rate of 0.4 mL/min. The gradient elution was as follows: 1–40% B (0–11 min), 40–70% B (11–13 min), and 70–100% B (13–15 min), followed by column washing and re-equilibration.

Mass spectrometry was performed in both positive and negative ionisation modes using data-dependent acquisition. MS1 scans were acquired over an *m*/*z* range of 50–1200, and precursor ions exceeding a defined intensity threshold were selected for tandem mass spectrometry (MS/MS) fragmentation with dynamic exclusion applied. Samples were analysed in triplicate along with solvent blanks.

Raw UPLC-MS data were processed using MS-DIAL (v. 5.2; RIKEN Center for Sustainable Resource Science, Yokohama, Japan) [12,13] for peak detection, alignment, and annotation. Phytochemical features were tentatively annotated by matching MS/MS spectra against public spectral libraries, including the RIKEN public spectral library and the Global Natural Products Social Molecular Networking (GNPS) database [14]. Data filtering was conducted using MS-CleanR (v. 1.0; Université de Toulouse, Toulouse, France) [15] to remove background and low-quality features. Selected metabolite annotations were further supported using SIRIUS (v. 5.8; Friedrich Schiller University Jena, Jena, Germany) [16]. Because authentic reference standards were not used, all phytochemical feature assignments were considered tentative and interpreted according to accepted metabolomics reporting and high-resolution MS annotation guidance [17,18]. Multivariate group-separation analyses performed using MetaboAnalyst 5.0 were not included because the UPLC-QTOF-MS analysis was used primarily for phytochemical profiling of the JGF extract rather than comparison of multiple biological treatment groups.

### 2.3. Cell Culture

Murine macrophage RAW 264.7 cells (ATCC, Manassas, VA, USA) were cultured in DMEM containing 4.5 g/L glucose, L-glutamine, and sodium pyruvate. The complete growth medium was supplemented with 5% foetal bovine serum (FBS; Interpath Services, Somerton, VIC, Australia) and 100 U/mL penicillin and 100 µg/mL streptomycin (Sigma-Aldrich, St. Louis, MO, USA). Cells were maintained in a humidified incubator at 37 °C with 5% CO_2_ and subcultured upon reaching 80–90% confluency. For experiments, cells were seeded into 96-well plates at 8.5 × 10^4^ cells/well in 100 µL medium and allowed to adhere for 45 h [19].

### 2.4. Measurement of Anti-Inflammatory Activity

Cells were pre-treated with the JGF extract at concentrations of 4.69–150 μg/mL or vehicle control for 2 h, followed by stimulation with lipopolysaccharide (LPS; *Escherichia coli* O111:B4; Sigma-Aldrich, St. Louis, MO, USA) at a final concentration of 50 ng/mL for 16 h. In parallel, 1400 W (Cayman Chemical, Ann Arbor, MI, USA), a selective inducible nitric oxide synthase (iNOS/NOS2) inhibitor, was used as a positive control over a concentration range of 0.123–30 µg/mL under the same experimental conditions. NO production was quantified by measuring nitrite accumulation in the culture supernatant using the Griess assay [20]. Briefly, 100 µL of cell culture supernatant was mixed with 100 µL of freshly prepared Griess reagent (1% sulfanilamide in 5% phosphoric acid and 0.1% N-(1-naphthyl) ethylenediamine dihydrochloride). Absorbance was measured at 540 nm using a CLARIOstar microplate reader (BMG LABTECH Pty. Ltd., Mornington, VIC, Australia). Nitrite concentrations were calculated from a sodium nitrite standard curve and expressed relative to LPS-stimulated control cells.

To assess cytotoxicity, a 3-(4,5-dimethylthiazol-2-yl)-2,5-diphenyltetrazolium bromide (MTT) assay was performed in parallel. Following removal of supernatants, fresh serum-free medium containing 0.12 mg/mL MTT was added and incubated for 2 h at 37 °C. The medium was then removed, and formazan crystals were dissolved in 100 µL of dimethyl sulfoxide (DMSO). Absorbance was measured at 510 nm using the same microplate reader. Cell viability was expressed as a percentage relative to LPS-stimulated control cells.

### 2.5. Quantification of Pro-Inflammatory Cytokines

The concentrations of tumour necrosis factor-alpha (TNF-α) and interleukin-6 (IL-6) were quantified in aliquots of the same cell culture supernatants collected under the treatment conditions described in Section 2.4, using commercially available sandwich enzyme-linked immunosorbent assay (ELISA) kits (TNF-α: Cat. No. 900-K54; IL-6: Cat. No. 900-K50; PeproTech, Rocky Hill, NJ, USA) according to the manufacturer’s instructions. Overall sample dilution factors of 1:20 and 1:200 were applied to the IL-6 and TNF-α assays, respectively.

Briefly, high-binding 96-well microplates (Corning Incorporated, Corning, NY, USA; Cat. No. 3590) were coated overnight at room temperature with capture antibodies diluted in carbonate-bicarbonate buffer (pH 9.5) and blocked with 2% bovine serum albumin (BSA) in PBS. Standards and samples were added and incubated for 2 h at room temperature, followed by incubation with biotinylated detection antibodies and streptavidin–horseradish peroxidase (HRP).

Signal development was performed using 3,3′,5,5′-tetramethylbenzidine (TMB) substrate (Sigma-Aldrich, St. Louis, MO, USA; Cat. No. T8665), and the reaction was stopped with 0.5 M sulfuric acid. Absorbance was measured at 450 nm with wavelength correction at 570 nm using a CLARIOstar microplate reader. Absolute cytokine concentrations were interpolated from assay-specific standard curves and corrected for the respective sample dilution factors. Relative cytokine release was normalised to the unstimulated and LPS-stimulated controls, defined as 0% and 100%, respectively. Unstimulated cells served as negative controls, whereas LPS-stimulated cells served as positive controls. Measurements for all experimental groups were obtained from three independent biological experiments, with technical replicate measurements averaged within each experiment before analysis.

### 2.6. Proteomic Analysis by Label-Free Quantification

RAW 264.7 cells were seeded in 6-well plates at a density of 8.5 × 10^5^ cells/mL (2 mL/well) and cultured for 48 h. The medium was replaced with serum-free DMEM, and cells were treated in triplicate with JGF extract (100 µg/mL) for 2 h, followed by stimulation with LPS (50 ng/mL) for 24 h. Untreated cells served as negative controls, and LPS-stimulated cells served as positive controls.

For proteomic analysis, both conditioned media (secretome) and cellular lysates were collected. Culture media were collected, freeze-dried, and reconstituted in lysis buffer. Cells were harvested, washed with ice-cold PBS, and lysed on ice using a mass spectrometry-compatible lysis buffer from the EasyPep™ Mini MS Sample Prep Kit (Thermo Fisher Scientific, Waltham, MA, USA; Cat. No. A40006), supplemented with nuclease and protease/phosphatase inhibitors. Insoluble debris was removed by centrifugation, and the supernatant containing total protein was collected. Protein concentrations were determined using a bicinchoninic acid (BCA) assay with the Pierce™ Rapid Gold BCA Protein Assay Kit (Thermo Fisher Scientific, Waltham, MA, USA; Cat. No. A53225). For each sample, 100 µg of protein was subjected to tryptic digestion using the EasyPep™ Mini MS Sample Prep Kit. Proteins were reduced, alkylated, and digested with trypsin/Lys-C at 37 °C. Peptides were purified, dried, and reconstituted in 0.1% formic acid.

Peptides were analysed using a nanoACQUITY UPLC system coupled to a SYNAPT G2-S HDMS mass spectrometer (Waters Corporation, Milford, MA, USA) operating in MS^E^ data-independent acquisition mode. Separation was performed on a nanoEase M/Z BEH C18 column (Waters Corporation, Milford, MA, USA) using a gradient of water and acetonitrile containing 0.1% formic acid. Raw data were processed using Progenesis QI for Proteomics (Nonlinear Dynamics, Newcastle upon Tyne, UK) and searched against the UniProt *Mus musculus* database. Proteins were considered differentially expressed based on one-way analysis of variance (ANOVA) with a false discovery rate (FDR)-adjusted *p*-value ≤ 0.05 and an absolute log_2_ fold change ≥0.58.

Functional pathway analysis was performed using Ingenuity Pathway Analysis (IPA; QIAGEN, Redwood City, CA, USA). IPA was performed separately for the cellular proteome and secretome datasets. Because the secretome dataset contained fewer quantified proteins, a more conservative absolute activation Z-score threshold of ≥1 was used to restrict the secretome visualisation to stronger pathway predictions, whereas an absolute activation Z-score threshold of ≥0.5 was used to summarise the broader cellular proteome dataset. Both analyses additionally required a Benjamini–Hochberg-adjusted *p*-value (Q ≤ 0.05). These dataset-specific thresholds were used only to prioritise pathways for visualisation, and the cellular proteome and secretome IPA outputs were not directly compared quantitatively. This label-free proteomic analysis was used as a comparative, discovery-level approach to identify treatment-associated protein changes.

Mass spectrometry proteomics data were deposited in the ProteomeXchange Consortium via the PRIDE repository under the dataset identifier PXD066295 (DOI: 10.6019/PXD066295).

### 2.7. Statistical Analysis

All experiments were performed with at least three independent biological replicates. Data are presented as the mean ± standard deviation (SD) or standard error of the mean (SEM), as indicated in the relevant figure legends and table footnotes. Statistical analyses were conducted using GraphPad Prism (version 10.1.2, GraphPad Software, San Diego, CA, USA). Concentration–response curves and descriptive half-maximal inhibitory concentration (IC_50_) estimates for JGF and 1400W in the NO assay were obtained using four-parameter nonlinear regression with a variable slope. For inferential analysis of JGF-mediated NO production, technical triplicates were averaged within each of three independent biological experiments, and the experiment-level means were analysed using one-way repeated-measures ANOVA followed by Dunnett’s multiple-comparisons test, with each JGF concentration compared with the LPS-stimulated control. Statistical procedures used for the proteomic analyses are described in Section 2.6. A *p*-value < 0.05 was considered statistically significant.

## 3. Results and Discussion

### 3.1. Phytochemical Profiling of JGF Reveals Predominant Flavonoid- and Phenolic-Related Annotated Features

To characterise the chemical composition of Jing Guan Fang (JGF), UPLC-QTOF-MS-based phytochemical profiling was performed in both positive and negative ionisation modes. The analysis revealed a chemically diverse profile of phytochemical features, with many prominent annotations corresponding to flavonoid- and phenolic-related compounds, together with selected terpenoid- and alkaloid-related annotations. A representative set of significant annotated features is shown in Table 1, arranged by ascending retention time, while the full list of abundant significant features is provided in Supplementary Table S1.

Flavonoid-related annotations were prominent within the JGF chemical profile, including features assigned to flavones, flavonols, chalcones, and glycosylated derivatives. Representative features included those tentatively assigned as baicalein, wogonin, chrysin, luteolin-related derivatives, and quercetin. Phenolic-related annotations were also observed, including honokiol, which is classified as a lignan-type phenolic compound rather than a terpenoid. Features corresponding to selected terpenoid- and alkaloid-related compounds were also present, although these were less predominant than the flavonoid- and phenolic-related annotations shown in Table 1.

Several of the tentatively annotated compounds, including baicalein and wogonin, have previously been reported to exhibit anti-inflammatory effects in vitro and in vivo, including in LPS-stimulated RAW 264.7 macrophages, supporting the biological relevance of these JGF-associated phytochemical features [21,22]. However, because authentic reference standards were not used in the present study, these assignments should be interpreted as tentative annotations rather than confirmed compound identifications.

The predominance of flavonoid- and phenolic-related annotated features is relevant to the scope of this study, as many compounds within these chemical classes have been associated with anti-inflammatory and antioxidant activities [23,24]. This phytochemical profile provides a compositional context for the observed modulation of macrophage inflammatory responses and oxidative stress-related signalling pathways examined in the subsequent functional and proteomic analyses.

SwissADME (SIB Swiss Institute of Bioinformatics, Molecular Modeling Group, Lausanne, Switzerland) was used to further assess tentative compound annotations corresponding to selected abundant features with respect to predicted gastrointestinal absorption, blood–brain barrier permeability, and P-glycoprotein substrate status (Figure S1 and Supplementary Table S2). Several tentative compound annotations, including baicalein, wogonin, chrysin, and honokiol, were predicted to have high gastrointestinal absorption. Several were also predicted not to be P-glycoprotein substrates, whereas predicted blood–brain barrier permeability varied among the selected annotations and should be interpreted cautiously in relation to systemic versus neuroinflammatory relevance.

### 3.2. JGF Reduces LPS-Induced Inflammatory Mediator Production Without Reducing Cell Viability

**Figure 1:**
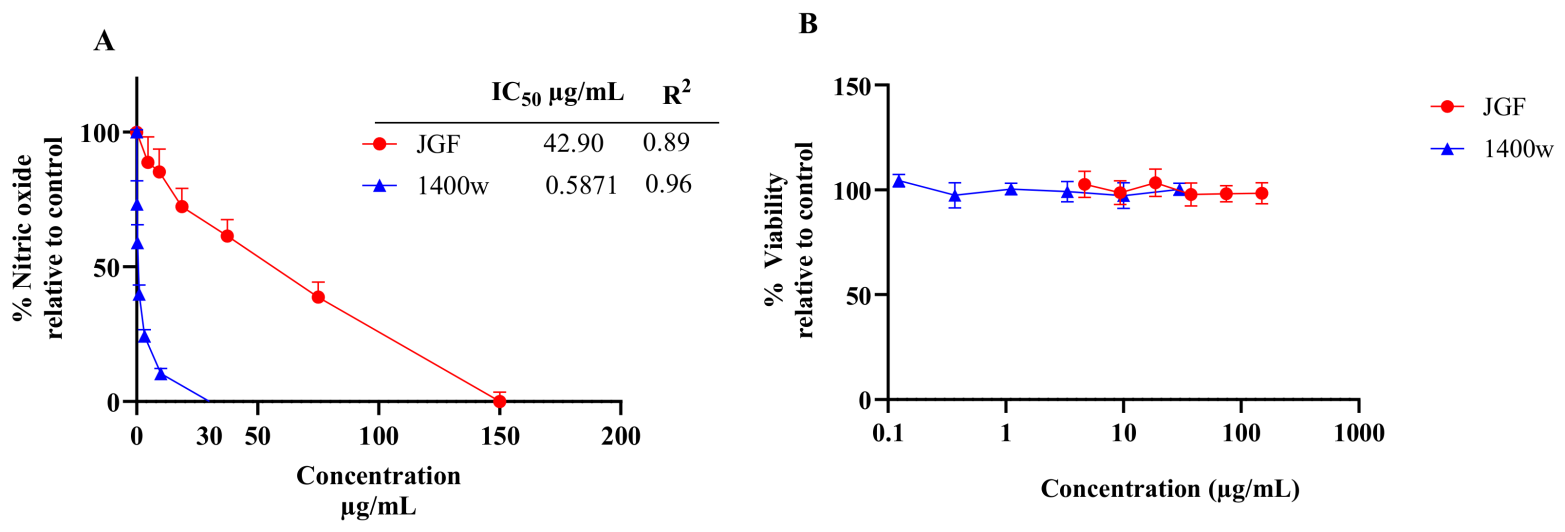
JGF reduces nitric oxide production without reducing cell viability in LPS-stimulated RAW 264.7 macrophages. (**A**) Nitric oxide (NO) production following treatment with JGF and 1400W, a selective iNOS/NOS2 inhibitor used as a positive control for inhibition of LPS-induced NO production. Concentration–response curves and the corresponding IC_50_ and R^2^ values were obtained using four-parameter nonlinear regression with a variable slope and are presented as descriptive summaries. (**B**) Cell viability of RAW 264.7 cells following treatment, assessed using the MTT assay. Data are presented as mean ± SD from three independent biological experiments, each performed in technical triplicate. For inferential analysis of JGF-mediated NO inhibition, technical triplicates were averaged within each experiment, and the experiment-level means were analysed using one-way repeated-measures ANOVA followed by Dunnett’s multiple-comparisons test against the LPS-stimulated control. The overall effect of JGF concentration on NO production was significant (*F*(6, 12) = 7.81, *p* = 0.0014), with significant reductions at 150 µg/mL (adjusted *p* = 0.0011), 75 µg/mL (adjusted *p* = 0.0225), and 9.38 µg/mL (adjusted *p* = 0.0354); the remaining concentrations were not statistically significant. Abbreviations: JGF, Jing Guan Fang; LPS, lipopolysaccharide; iNOS, inducible nitric oxide synthase; NOS2, nitric oxide synthase 2; IC_50_, half-maximal inhibitory concentration; R^2^, coefficient of determination; MTT, 3-(4,5-dimethylthiazol-2-yl)-2,5-diphenyltetrazolium bromide; SD, standard deviation; ANOVA, analysis of variance.

The anti-inflammatory activity of JGF was evaluated in LPS-stimulated RAW 264.7 macrophages by measuring NO production, cell viability, and pro-inflammatory cytokine release (Figure 1 and Table 2) [25]. A significant overall effect of JGF concentration on NO production was observed, with significant reductions at 150, 75, and 9.38 µg/mL compared with the LPS-stimulated control; the remaining concentrations were not statistically significant (Figure 1A).

Importantly, JGF did not reduce cell viability across the tested concentrations, indicating that the observed reductions in NO production were not attributable to overt cytotoxicity (Figure 1B). This finding supports the interpretation that the reduction in inflammatory mediator production was not caused by impaired cell survival. JGF also reduced pro-inflammatory cytokine release, with a clear concentration-dependent reduction in IL-6 release, whereas the TNF-α response was more variable across the tested concentrations (Table 2).

IL-6 release decreased progressively with increasing JGF concentration. In contrast, TNF-α release showed greater inter-experiment variability and no consistently graded concentration-response pattern across the tested concentrations. Because TNF-α release did not reach 50% inhibition relative to the LPS-stimulated control within the tested concentration range, a reliable IC_50_ estimate could not be obtained. The basis for the difference between the IL-6 and TNF-α responses cannot be established from the present single-time-point dataset and requires further investigation. Collectively, these findings suggest that JGF reduces LPS-induced inflammatory mediator production while maintaining cellular viability within the concentration range assessed for cytotoxicity.

### 3.3. JGF Is Associated with Modulation of LPS-Induced Cellular Proteomic Alterations in Macrophages

**Figure 2:**
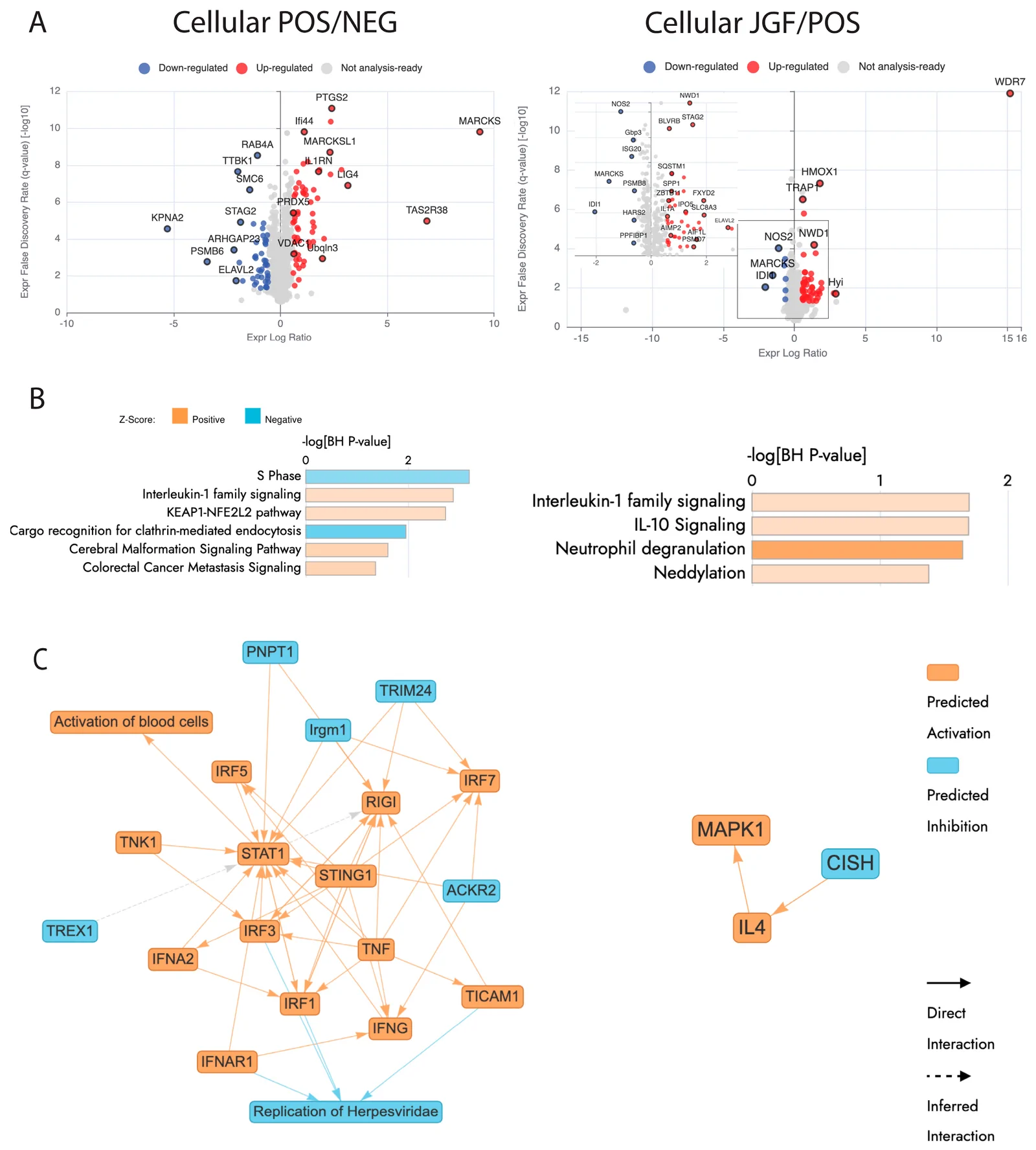
Discovery-level proteomic analysis of RAW 264.7 cell lysates under inflammatory stimulation and JGF treatment. (**A**) Volcano plots of differentially expressed proteins (DEPs) comparing LPS-stimulated cells with the negative control (POS/NEG, **left**) and JGF-treated LPS-stimulated cells with the LPS-only control (JGF/POS, **right**). DEPs were identified using a Q-value ≤ 0.05 and absolute log_2_ fold change ≥ 0.58. (**B**) Significantly enriched canonical pathways identified via exploratory Ingenuity Pathway Analysis (IPA) based on DEPs from the same comparisons: POS/NEG (**left**) and JGF/POS (**right**), using a Benjamini–Hochberg-adjusted *p*-value (Q ≤ 0.05) and an absolute activation Z-score ≥ 0.5. (**C**) IPA graphical summaries of predicted upstream and downstream effects and regulatory networks for POS/NEG (**left**) and JGF/POS (**right**). Predicted activation is shown in orange, predicted inhibition in blue, and molecular interactions as solid (direct) or dashed (indirect) lines. The cellular proteome IPA output was analysed independently of the secretome output, and the pathway thresholds were used only to prioritise pathways for visualisation. Abbreviations: JGF, Jing Guan Fang; LPS, lipopolysaccharide.

Label-free quantitative proteomic analysis was performed to investigate treatment-associated protein changes in JGF-treated, LPS-stimulated RAW 264.7 macrophages (Figure 2 and Table 3). For proteomic analysis, JGF was applied at 100 µg/mL, a concentration within the non-cytotoxic range established in the functional assays. LPS stimulation induced substantial alterations in cellular protein expression profiles associated with inflammatory signalling, whereas JGF treatment was associated with partial modulation of these LPS-induced proteomic changes.

JGF treatment was associated with reduced abundance of selected inflammation-associated proteins, including MARCKS and NOS2 (Table 3). Detailed per-replicate normalised MARCKS abundance values, together with peptide-count and statistical information, are provided in Supplementary File S1 (worksheet “Table S2”). Because MARCKS abundance was very low in the negative-control group, the large POS_C/NEG_C fold-change estimate should be interpreted cautiously. MARCKS is involved in cytoskeletal regulation and inflammatory signalling [26], while NOS2 is a major mediator of inducible NO production during macrophage activation. The reduced abundance of NOS2 is consistent with, but does not alone confirm, the observed reduction in NO production described in Section 3.2.

In addition to changes in inflammation-associated proteins, JGF treatment was associated with restoration of selected regulatory proteins, including AIMP1 and AIMP2. AIMP2 has been implicated in TNF-α/TRAF2-related signalling [27,28], suggesting a potential association between JGF-related protein changes and endogenous inflammatory regulatory pathways. These findings should be interpreted as discovery-level proteomic observations and require further validation using targeted approaches.

Together, these cellular proteomic findings provide discovery-level molecular support for the functional anti-inflammatory effects observed in Section 3.2. The reduced abundance of NOS2 is consistent with decreased NO production, while MARCKS downregulation suggests treatment-associated modulation of inflammation-related cytoskeletal and signalling responses. In parallel, restoration of AIMP1 and AIMP2 suggests potential involvement of selected endogenous regulatory pathways. Overall, these findings indicate that JGF treatment is associated with partial modulation of LPS-induced proteomic dysregulation in macrophages.

### 3.4. Secretome Proteomics Reveals JGF-Mediated Modulation of Extracellular Inflammatory Signalling

**Figure 3:**
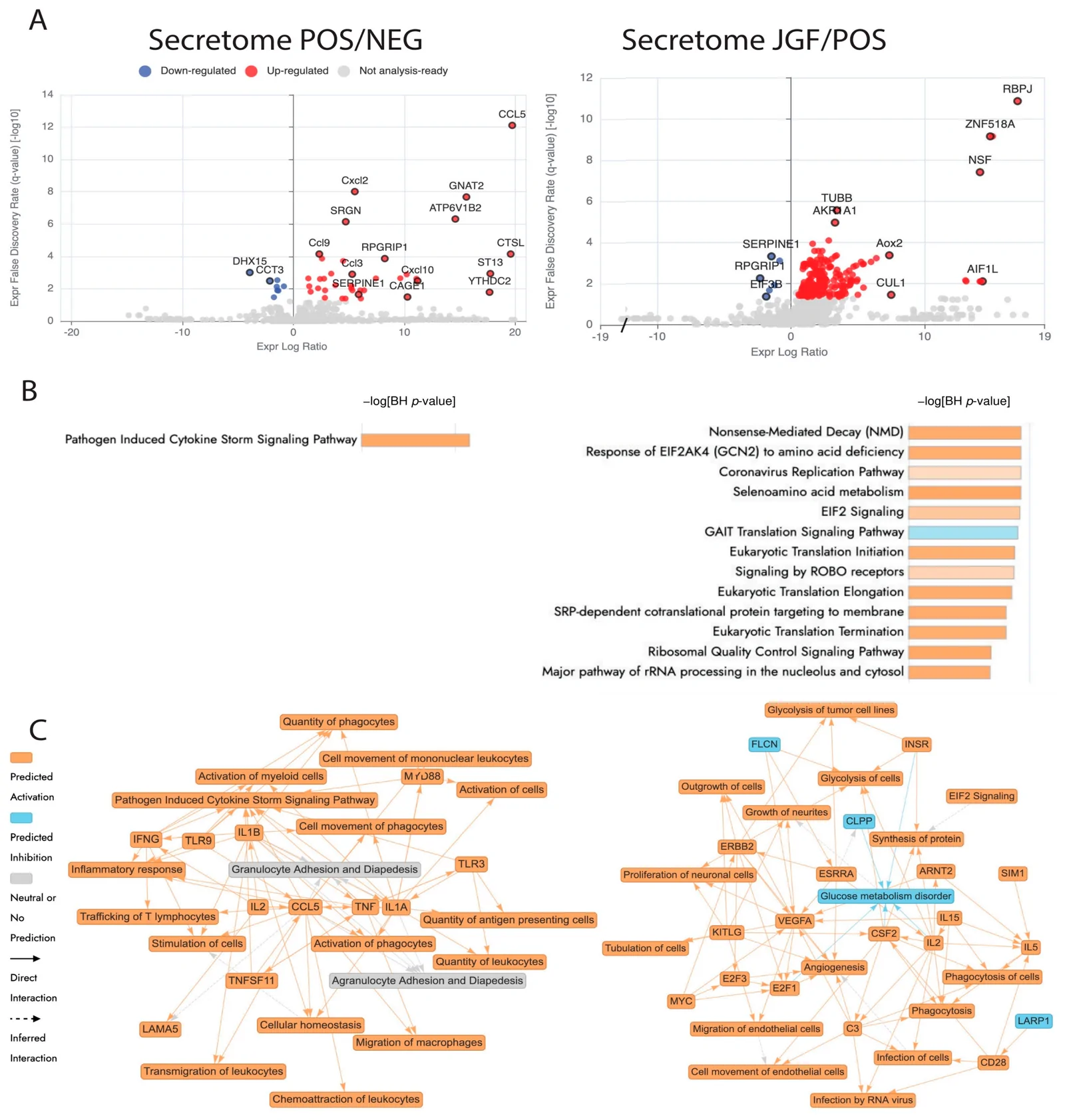
Secretome proteomics analysis reveals extracellular signalling changes in RAW 264.7 macrophages following LPS stimulation and JGF treatment. (**A**) Volcano plots showing differentially expressed secreted proteins in LPS-stimulated cells compared with negative control cells (Secretome POS/NEG, **left**) and JGF-treated LPS-stimulated cells compared with LPS-only cells (Secretome JGF/POS, **right**). Proteins were considered differentially expressed using a Q-value ≤ 0.05 and an absolute log_2_ fold change ≥ 0.58. (**B**) Significantly enriched canonical pathways identified by Ingenuity Pathway Analysis (IPA) for the POS/NEG (**left**) and JGF/POS (**right**) comparisons, based on Benjamini–Hochberg-adjusted *p*-value (Q ≤ 0.05) and absolute activation Z-score ≥ 1; for JGF/POS, only the top 13 pathways are shown. (**C**) IPA graphical summaries showing predicted biological functions and interaction networks across comparisons. Because the secretome dataset contained fewer quantified proteins, the more conservative absolute activation Z-score threshold of ≥1 was used to restrict the visualisation to stronger predicted pathway changes. The secretome IPA output was interpreted independently of, and was not directly compared quantitatively with the cellular proteome IPA output. Abbreviations: JGF, Jing Guan Fang; LPS, lipopolysaccharide.

To further investigate whether JGF modulates extracellular inflammatory signalling, secretome proteomic analysis was performed in LPS-stimulated RAW 264.7 macrophages following JGF treatment (Figure 3 and Table 4) [29]. Differentially expressed secreted proteins were identified between LPS-stimulated cells and untreated controls, as well as between JGF-treated LPS-stimulated cells and LPS-only controls.

Secretome analysis indicated that JGF modulated the extracellular inflammatory environment, with several secreted proteins showing reversal of LPS-induced changes (Table 4). Notably, JGF reduced the secretion of SERPINE1/PAI-1, a mediator linked to inflammatory, pro-fibrotic, and pro-thrombotic responses [30,31]. This finding suggests that JGF may regulate not only intracellular inflammatory signalling but also extracellular mediators associated with inflammation-related tissue remodelling.

Together, these secretome findings suggest that JGF modifies extracellular signalling responses associated with macrophage activation. The reduction in SERPINE1/PAI-1 secretion provides a secretome-level correlate to the intracellular anti-inflammatory effects observed in the cellular proteomic analysis, supporting a coordinated effect of JGF on both intracellular and extracellular inflammatory networks.

### 3.5. Overall Interpretation of JGF-Mediated Anti-Inflammatory Effects

The combined phytochemical, functional, cellular proteomic, and secretome analyses provide a discovery-level interpretation of the anti-inflammatory effects of JGF in LPS-stimulated macrophages. Phytochemical profiling revealed a chemically diverse profile characterised predominantly by flavonoid- and phenolic-related annotated features, together with selected terpenoid- and alkaloid-related annotations supporting the multi-component nature of the formula. Functionally, JGF reduced NO production and pro-inflammatory cytokine release without reducing cell viability, indicating anti-inflammatory activity that was not attributable to cytotoxicity.

At the intracellular level, cellular proteomics showed that JGF treatment was associated with modulation of LPS-induced protein dysregulation, including reduced abundance of inflammation-associated proteins such as MARCKS and NOS2. The reduced abundance of NOS2 is consistent with, but does not alone confirm, the observed decrease in NO production, while restoration of AIMP1 and AIMP2 suggests potential involvement of selected endogenous regulatory pathways involved in immune homeostasis.

At the extracellular level, secretome analysis further suggested that JGF treatment was associated with altered extracellular inflammatory signalling, including reduced secretion of SERPINE1/PAI-1, a mediator associated with inflammatory, pro-fibrotic, and pro-thrombotic responses. Together, these findings suggest that JGF is associated with modulation of inflammatory mediator production, selected intracellular protein changes, and extracellular secretome alterations in LPS-stimulated macrophages. These observations provide a discovery-level molecular basis for further targeted validation of JGF as a candidate multi-component anti-inflammatory formula.

### 3.6. Study Limitations and Future Directions

This study has several limitations. First, the UPLC-QTOF-MS analysis was used for phytochemical profiling of the JGF extract rather than for targeted quantitative metabolite confirmation. The phytochemical features reported in this study were based on tentative LC-MS/MS annotations using public spectral libraries and in silico annotation tools, without confirmation using authentic reference standards. Therefore, phytochemical feature assignments and relative abundance estimates should be interpreted cautiously as tentative and semi-quantitative, in line with accepted metabolomics reporting and high-resolution MS annotation guidance [17,18]. Future studies incorporating authentic reference standards and targeted quantitative LC-MS methods will be required to confirm and quantify key JGF constituents.

Second, the cellular proteomic and secretome analyses were designed as discovery-level approaches to identify treatment-associated protein changes rather than to establish validated mechanisms of action. Key proteins highlighted in this study, including NOS2, MARCKS, AIMP1/AIMP2, and SERPINE1/PAI-1, were not independently validated using orthogonal methods such as Western blotting, reverse transcription quantitative polymerase chain reaction (RT-qPCR), ELISA, or targeted proteomics. In particular, the large log_2_ fold change observed for MARCKS should be interpreted cautiously, because MARCKS abundance was very low in the negative-control group and not all data-processing steps could be independently verified from the available documentation. Future studies should validate these proteins using targeted approaches and employ functional perturbation experiments, such as small interfering RNA (siRNA)-mediated knockdown, pharmacological inhibition, or overexpression, to determine whether they are causally involved in the anti-inflammatory effects of JGF.

Third, secretome analysis was performed under serum-free conditions, which may influence basal secretome composition and stress-related protein release in RAW 264.7 macrophages. Therefore, the secretome findings should be interpreted as exploratory and treatment-associated rather than definitive evidence of extracellular mechanistic regulation. Future studies comparing serum-free, low-serum, and shorter collection conditions would help distinguish treatment-specific secretome changes from culture-condition-associated effects.

Fourth, the functional inflammatory assessment was limited to NO, IL-6, TNF-α, and cell viability. Although these are widely used inflammatory readouts in LPS-stimulated RAW 264.7 macrophages, they do not fully capture the broader cytokine and inflammatory mediator network involved in macrophage activation. Future studies using broader cytokine profiling, time-course analyses, and targeted pathway validation would strengthen the functional interpretation.

Finally, pathway analysis using Ingenuity Pathway Analysis should be interpreted as hypothesis-generating. IPA-based pathway and network predictions do not confirm pathway activation or inhibition without further experimental validation. In addition, this study was performed using an in vitro LPS-stimulated RAW 264.7 macrophage model. Although RAW 264.7 macrophages are widely used for investigating LPS-induced inflammatory responses, immortalised macrophage cell lines may differ from primary macrophages in selected functional and molecular responses [32,33]. Therefore, future studies using primary macrophages, human monocyte-derived macrophages, and in vivo inflammatory models will be required to confirm the translational relevance of these findings.

## 4. Conclusions

This study provides a combined phytochemical, functional, cellular proteomic, and secretome-based assessment of the anti-inflammatory effects of Jing Guan Fang in LPS-stimulated RAW 264.7 macrophages. UPLC-QTOF-MS-based phytochemical profiling revealed a chemically diverse formula characterised predominantly by flavonoid- and phenolic-related annotated features, together with selected terpenoid- and alkaloid-related annotations, supporting the multi-component nature of JGF. Functionally, JGF reduced NO production and pro-inflammatory cytokine release without inducing cytotoxicity.

Discovery-level proteomic analyses further suggested that JGF treatment was associated with modulation of LPS-induced inflammatory protein changes. At the cellular level, JGF reduced the abundance of selected inflammation-associated proteins, including MARCKS and NOS2, while restoring selected regulatory proteins such as AIMP1 and AIMP2. At the secretome level, JGF reduced SERPINE1/PAI-1 secretion, suggesting treatment-associated changes in extracellular inflammatory and tissue-remodelling-related mediators. Collectively, these findings provide complementary discovery-level phytochemical and molecular insight into the anti-inflammatory effects of JGF in LPS-stimulated macrophages. Future studies incorporating targeted phytochemical confirmation, molecular validation, broader cytokine profiling, and in vivo models will be important to further establish the translational relevance of these findings.

## Figures and Tables

**Table 1:** Representative significant phytochemical features in JGF compared with vehicle solvent, tentatively annotated by LC-MS in positive and negative ionisation modes and arranged by ascending retention time.

Average RT (min)	Average *m*/*z* (Da)	Tentative ID	Adduct Type	Formula	Ontology/Class	Relative %
4.73	328.1496	L-Sinoacutine #	[M+H]^+^	C_19_H_21_NO_4_	Phenanthrenes and derivatives	0.56
5.80	609.1587	Luteolin-7,3′-di-o-glucoside	[M−H]^−^	C_27_H_30_O_16_	Flavonoid-O-glycosides	0.69
6.12	301.0294	Quercetin #	[M−H]^−^	C_15_H_10_O_7_	Flavonols	0.20
6.19	609.1215	Quercetin 3-(2″-P-Coumarylglucoside)	[M−H]^−^	C_30_H_26_O_14_	Flavonoid-glycosides	0.25
7.38	285.0345	Luteolin	[M−H]^−^	C_15_H_10_O_6_	Flavones	0.29
8.95	315.0452	Isorhamnetin	[M−H]^−^	C_16_H_12_O_7_	Flavonols	0.30
9.40	285.0717	7-O-Methylbaicalein	[M+H]^+^	C_16_H_12_O_5_	7-O-methylated flavonoids	0.45
10.02	299.0439	Hispidulin	[M−H]^−^	C_16_H_12_O_6_	6-O-methylated flavonoids	0.49
10.32	269.0356	3,7,4′-Trihydroxyflavone	[M−H]^−^	C_15_H_10_O_5_	Flavonols	1.11
10.58	299.0512	Kaempferol-4′-methyl ether	[M−H]^−^	C_16_H_12_O_6_	Flavonols	0.25
10.70	269.0356	Baicalein	[M−H]^−^	C_15_H_10_O_5_	Flavones	1.24
12.07	253.0774	2′-Hydroxy-4-methoxychalcone #	[M−H]^−^	C_16_H_14_O_3_	2′-Hydroxychalcones	0.83
12.45	285.0675	Wogonin	[M+H]^+^	C_16_H_12_O_5_	8-O-methylated flavonoids	0.84
12.49	253.0407	Chrysin #	[M−H]^−^	C_15_H_10_O_4_	Flavones	0.76
12.61	269.0703	7-O-Methylchrysin	[M−H]^−^	C_15_H_10_O_5_	Flavonols	0.28
12.64	279.0902	2′,4′-Dihydroxychalcone	[M−H]^−^	C_15_H_20_O_5_	Chalcones	0.33
13.31	345.0913	Berberrubine #	[M+Na]^+^	C_19_H_16_NO_4_	Protoberberine alkaloids	0.35
13.86	265.1134	Honokiol #	[M−H]^−^	C_18_H_18_O_2_	Biphenyls and derivatives	0.88
14.18	359.1071	Gardenin B	[M+H]^+^	C_19_H_18_O_7_	Flavones	0.44

The relative percentage was calculated by dividing the feature intensity by the total intensity of all significant features compared with the blank, using a threshold of |log_2_FC| > 1 and Q-value < 0.05. Feature annotations are reported as tentative LC-MS/MS-based annotations and should not be interpreted as confirmed compound identifications. The full list of abundant significant phytochemical features, including unknown features, duplicated annotations, and features with lower MS/MS matching confidence, is provided in Supplementary Table S1. Features marked with # indicate selected abundant features prioritised for further SwissADME-based interpretation based on predicted gastrointestinal absorption, blood–brain barrier permeability, and P-glycoprotein substrate status. Abbreviations: JGF, Jing Guan Fang; LC-MS, liquid chromatography–mass spectrometry; RT, retention time; ID, identification; log_2_FC, log_2_ fold change; LC-MS/MS, liquid chromatography–tandem mass spectrometry.

**Table 2:** Effects of JGF on absolute concentrations and relative release of IL-6 and TNF-α in LPS-stimulated RAW 264.7 macrophages.

Treatment/JGF Concentration (µg/mL)	IL-6 Concentration (pg/mL)	IL-6 Relative Release (% of LPS Control)	TNF-α Concentration (pg/mL)	TNF-α Relative Release (% of LPS Control)
Unstimulated control	26.4 ± 1.5	0	968.3 ± 92.8	0
LPS-stimulated control	1634.5 ± 308.5	100	14,782.3 ± 912.6	100
150	166.8 ± 92.6	8.33 ± 3.94	9928.8 ± 3079.4	64.87 ± 22.29
75	513.6 ± 197.7	29.80 ± 7.20	12,707.3 ± 4908.5	84.98 ± 35.53
37.5	999.3 ± 355.8	59.71 ± 11.56	14,610.3 ± 4775.2	98.76 ± 34.57
18.75	1287.2 ± 465.5	77.53 ± 17.15	14,904.4 ± 3692.9	100.88 ± 26.73
9.38	1403.1 ± 448.5	84.94 ± 15.52	12,495.3 ± 2545.1	83.44 ± 18.42
4.69	1512.2 ± 224.4	93.64 ± 14.99	16,401.2 ± 1277.4	111.72 ± 9.25

Data are presented as mean ± SD from three independent biological experiments (*n* = 3), with technical replicates averaged within each experiment. Absolute cytokine concentrations are reported in pg/mL. Relative cytokine release was calculated within each biological experiment by setting the corresponding unstimulated and LPS-stimulated controls to 0% and 100%, respectively, and the resulting percentages were then averaged across the three biological experiments. Abbreviations: JGF, Jing Guan Fang; IL-6, interleukin-6; TNF-α, tumour necrosis factor-α; LPS, lipopolysaccharide; SD, standard deviation.

**Table 3:** Significantly regulated proteins identified in the cellular lysate of RAW 264.7 macrophages. The table shows the log_2_ fold change (log_2_FC) for proteins comparing LPS-stimulated cells to the negative control (POS_C/NEG_C) and comparing JGF-treated cells to the LPS-stimulated control (JGF_C/POS_C). Regulation reversal indicates whether JGF treatment induced an expression change in the opposite direction to that caused by LPS stimulation (Y = Yes; N = No). All listed proteins were significantly regulated with an adjusted *p*-value ≤ 0.05 and an absolute log_2_ fold change ≥ 0.58.

Gene Symbol	Description	log_2_FC (POS_C/NEG_C)	log_2_FC (JGF_C/POS_C)	Regulation Reversal
*Aif1l*	Allograft inflammatory factor 1-like	−1.29	1.65	Y
*Aimp1*	Aminoacyl tRNA synthase complex-interacting multifunctional protein 1	−1.16	0.65	Y
*Aimp2*	Aminoacyl tRNA synthase complex-interacting multifunctional protein 2	−1.01	0.71	Y
*Atp11a*	Phospholipid-transporting ATPase IH	−1.13	1.73	Y
*Elavl2*	ELAV-like protein 2	−2.08	2.78	Y
*Galk1*	Galactokinase	−0.89	1.11	Y
*Gars1*	Glycine--tRNA ligase	−0.71	0.61	Y
*Gbp3*	Guanylate-binding protein 3	1.82	−0.65	Y
*Get3*	ATPase GET3	−1.93	1.30	Y
*Gmfb*	Glia maturation factor beta	−1.06	1.78	Y
*Hnrnpul1*	Heterogeneous nuclear ribonucleoprotein U-like protein 1	1.17	0.75	N
*Hyi*	Hydroxypyruvate isomerase	−1.76	2.92	Y
*Isg20*	Interferon-stimulated gene 20 kDa protein	2.85	−0.70	Y
*Map2*	Microtubule-associated protein 2	−0.71	0.59	Y
*Marcks*	Myristoylated alanine-rich C-kinase substrate	9.33	−1.52	Y
*Nos2*	Nitric oxide synthase, inducible	1.56	−1.10	Y
*Nwd1*	NACHT domain- and WD repeat-containing protein 1	0.83	1.40	N
*Psmb8*	Proteasome subunit beta type-8	0.79	−0.61	Y
*Sqstm1*	Sequestosome-1	2.34	0.74	N
*Stag2*	Cohesin subunit SA-2	−1.87	1.50	Y
*Zbtb11*	Zinc finger and BTB domain-containing protein 11	−0.69	0.63	Y

Genes are arranged alphabetically. Abbreviations: JGF, Jing Guan Fang; LPS, lipopolysaccharide; NEG_C, negative control (cellular lysate); POS_C, LPS-stimulated control (cellular lysate); JGF_C, JGF-treated LPS-stimulated group (cellular lysate).

**Table 4:** Significantly regulated proteins identified in the secretome of RAW 264.7 macrophages. The table shows the log_2_ fold change (log_2_FC) for secreted proteins comparing LPS-stimulated cells to the negative control (POS_S/NEG_S) and comparing JGF-treated LPS-stimulated cells to the LPS-stimulated control (JGF_S/POS_S). Regulation reversal indicates whether JGF treatment induced an expression change in the opposite direction to that caused by LPS stimulation (Y = Yes; N = No). All listed proteins were significantly regulated with an adjusted *p*-value ≤ 0.05 and an absolute log_2_ fold change ≥ 0.58.

Gene Symbol	Description	log_2_FC (POS_S/NEG_S)	log_2_FC (JGF_S/POS_S)	Regulation Reversal
*Anp32b*	Acidic leucine-rich nuclear phosphoprotein 32 family member B	−0.86	1.56	Y
*Anxa2*	Annexin A2	1	−1.23	Y
*Apex1*	DNA repair nuclease/redox regulator APEX1	4.41	1.15	N
*Dhx15*	ATP-dependent RNA helicase DHX15	−3.93	2.39	Y
*Eif3b*	Eukaryotic translation initiation factor 3 subunit B	2.84	−1.85	Y
*Hnrnpk*	Heterogeneous nuclear ribonucleoprotein K	−1.46	0.91	Y
*Plxna3*	Plexin-A3	1.56	0.87	N
*Rpgrip1*	X-linked retinitis pigmentosa GTPase regulator-interacting protein 1	8.24	−2.31	Y
*Rpsa*	Small ribosomal subunit protein uS2	−1.44	1.08	Y
*Serpine1*	Plasminogen activator inhibitor 1	5.89	−1.46	Y

Genes are arranged alphabetically. Abbreviations: JGF, Jing Guan Fang; LPS, lipopolysaccharide; NEG_S, negative control (secretome); POS_S, LPS-stimulated control (secretome); JGF_S, JGF-treated LPS-stimulated group (secretome).

## Data Availability

The original contributions presented in this study are included in the article and Supplementary Materials. The mass spectrometry proteomics data have been deposited in the ProteomeXchange Consortium via the PRIDE repository under the dataset identifier PXD066295 (DOI: 10.6019/PXD066295). Other data supporting the findings of this study are available from the corresponding author upon reasonable request.

## Supplementary Materials

The following supporting information can be downloaded at: https://www.mdpi.com/article/10.3390/antiox15081010/s1; Figure S1: BOILED-Egg plot of selected abundant tentatively annotated JGF features; Table S1: Full list of abundant significant phytochemical features detected in JGF compared with vehicle solvent, tentatively annotated by LC-MS in positive and negative ionisation modes; Table S2: SwissADME-derived molecular ADME properties of selected abundant tentatively annotated JGF features; Supplementary File S1: Detailed LC-MS feature-level data and quantitative proteomics data, including per-replicate normalised MARCKS abundance values and associated peptide and statistical information.
