# Supplementary material for: Phytochemical Profiling and Proteomic Insights into the Anti-Inflammatory Effects of Jing Guan Fang in LPS-Stimulated Macrophages
Source: Antioxidants (Basel). 2026 Aug 13;15(8):1010. doi: 10.3390/antiox15081010 (PMC13509815; doi:10.3390/antiox15081010)
Supplement: Supplementary file 1 [file antioxidants-15-01010-s001.zip › antioxidants-4374140_revised_supplementary_Clean.pdf]

Supplementary Table S1. Full list of abundant significant phytochemical features in JGF compared with vehicle solvent, tentatively annotated by LC-MS in positive and negative ionisation modes.

Average RT (min)	Average m/z (Da)	Tentative ID	Adduct type	Formula	Ontology/Class	Relative %
**14.38**	265.1129	(E)-5-(4-methoxy-5-methyl-6-oxopyran-2-yl)-3-methylhex-4-enoic acid	$[M-H]^-$	$C_{14}H_{18}O_5$	Medium-chain fatty acids	1.27
**10.7**	269.0356	Baicalein	$[M-H]^-$	$C_{15}H_{10}O_5$	Flavones	1.24
**10.32**	269.0356	3,7,4'-Trihydroxyflavone	$[M-H]^-$	$C_{15}H_{10}O_5$	Flavonols	1.11
**12.45**	268.0266	Saccharin N-(2-acetic acid ethyl ester)	[M-H]-	$C_{11}H_{11}NO_5S$	Alpha amino acid esters	0.97
**12.82**	281.1058	11-Sulfoundecaneperoxoic acid	$[2M-2H+Na]^-$	$C_{11}H_{22}O_6S$	Benzenesulfonic acids and derivatives	0.89
**12.64**	373.0787	Baeomycesic acid	$[M-H]^-$	$C_{19}H_{18}O_8$	Depsides and depsidones	0.89
**13.86**	265.1134	Honokiol#	$[M-H]^-$	$C_{18}H_{18}O_2$	Biphenyls and derivatives	0.88
**12.45**	285.0675	Wogonin	$[M+H]^+$	$C_{16}H_{12}O_5$	8-O-methylated flavonoids	0.84
**12.07**	253.0774	2'-Hydroxy-4-methoxychalcone#	$[M-H]^-$	$C_{16}H_{14}O_3$	2'-Hydroxychalcones	0.83
**3.59**	461.1473	5,7-Dimethoxy-2-[4-[3,4,5-trihydroxy-6-(hydroxymethyl)oxan-2-yl]oxyphenyl]-2,3-dihydrochromen-4-one	$[M-H]^-$	$C_{23}H_{26}O_{10}$	Flavanones glycosides	0.82
**12.49**	253.0407	Chrysin#	$[M-H]^-$	$C_{15}H_{10}O_4$	Flavones	0.76
**5.8**	609.1587	Luteolin-7,3'-di-o-glucoside	$[M-H]^-$	$C_{27}H_{30}O_{16}$	Flavonoid-O-glycosides	0.69
**7.95**	345.0528	2-(3,4-dihydroxyphenyl)-5,7-dihydroxy-6,8-dimethoxychromen-4-one	$[M-H]^-$	$C_{17}H_{14}O_8$	Flavones	0.68
**4.12**	314.1708	Codeine-6-Methyl Ether	$[M+H]^+$	$C_{19}H_{23}NO_3$	Phenanthrenes and derivatives	0.68
**13.72**	333.1927	3,4,5-trihydroxy-6-(hydroxymethyl)oxan-2-yl decanoate	$[M-H]^-$	$C_{16}H_{30}O_7$	Fatty acyl glycosides of mono- and disaccharides	0.65

Average RT (min)	Average m/z (Da)	Tentative ID	Adduct type	Formula	Ontology/Class	Relative %
**8.87**	445.0601	CDP-ETHANOLAMINE	$[M-H]^-$	$C_{11}H_{20}N_4O_{11}P_2$	Nucleosides	0.62
**6.65**	547.1239	1-[(2S,3S)-3,5-dihydroxy-3,4-dihydro-2H-chromen-2-yl]-3,4,5-trihydroxy-8-[(2R)-3,5,7-trihydroxy-3,4-dihydro-2H-chromen-2-yl]benzo[7]annulen-6-one	$[M-H]^-$	$C_{29}H_{24}O_{11}$	Flavan-3-ols	0.57
**4.73**	328.1496	L-Sinoacutine#	$[M+H]^+$	$C_{19}H_{21}NO_4$	Phenanthrenes and derivatives	0.56
**3.22**	375.1181	(1S,4aS,7S,7aS)-7-hydroxy-7-methyl-1-[(2S,3R,4S,5S,6R)-3,4,5-trihydroxy-6-(hydroxymethyl)oxan-2-yl]oxy-4a,5,6,7a-tetrahydro-1H-cyclopenta[c]pyran-4-carboxylic acid	$[M-H]^-$	$C_{16}H_{24}O_{10}$	Iridoid O-glycosides	0.51
**10.02**	299.0439	Hispidulin	$[M-H]^-$	$C_{16}H_{12}O_6$	6-O-methylated flavonoids	0.49
**5.23**	303.0443	(2R,3R)-2-(2,6-dihydroxyphenyl)-3,5,7-trihydroxy-2,3-dihydrochromen-4-one	$[M-H]^-$	$C_{15}H_{12}O_7$	Flavanonols	0.49
**9.39**	919.1574	C50H32O18	$[M-H]^-$	null	null	0.46
**9.4**	285.0717	7-O-Methylbaicalein	$[M+H]^+$	$C_{16}H_{12}O_5$	7-O-methylated flavonoids	0.45
**14.18**	359.1071	Gardenin B	$[M+H]^+$	$C_{19}H_{18}O_7$	Flavones	0.44
**9.39**	459.0768	8-(7-Methoxycoumarin-6-yl) luteolin	$[M-H]^-$	$C_{25}H_{16}O_9$	Flavones	0.43
**13.31**	345.0913	Berberrubine#	$[M+Na]^+$	$C_{19}H_{16}NO_4$	Protoberberine alkaloids	0.35
**8.1**	269.0366	3,7,4'-Trihydroxyflavone	$[M-H]^-$	$C_{15}H_{10}O_5$	Flavonols	0.34
**12.64**	279.0902	2',4'-Dihydroxychalcone	$[M-H]^-$	$C_{15}H_{20}O_5$	Chalcones	0.33
**9.05**	285.0714	7-O-Methylbaicalein	$[M+H]^+$	$C_{16}H_{12}O_5$	7-O-methylated flavonoids	0.33
**9.16**	475.0689	6-(8''-Umbelliferyl)-Apigenin	$[M+FA-H]^-$	$C_{24}H_{14}O_8$	Flavones	0.32

**Average RT (min)**	**Average m/z (Da)**	**Tentative ID**	**Adduct type**	**Formula**	**Ontology/Class**	**Relative %**
**5.32**	639.1685	CID 122229730	$[M-H]^-$	$C_{32}H_{32}O_{14}$	Flavonoid-glycosides	0.31
**12.68**	825.4291	(2S,3S,4S,5R,6R)-6-[[(2S,3R,4R,6aR,6bS,8aS,14bR)-2-hydroxy-4-(hydroxymethyl)-4,6a,6b,11,11,14b-hexamethyl-8a-[(2S,3R,4S,5S,6R)-3,4,5-trihydroxy-6-(hydroxymethyl)oxan-2-yl]oxycarbonyl-1,2,3,4a,5,6,7,8,9,10,12,12a,14,14a-tetradecahydropicen-3-yl]oxy]-3,4,5-trihydroxyoxane-2-carboxylic acid	$[M-H]^-$	$C_{42}H_{66}O_{16}$	Triterpene saponins	0.31
**4.96**	297.1049	2-(2,3-dihydrobenzo[b][1,4]dioxin-6-yl)-6-methylchroman-4-one	$[M+H]^+$	$C_{18}H_{16}O_4$	Flavonolignans	0.31
**8.95**	315.0452	Isorhamnetin	$[M-H]^-$	$C_{16}H_{12}O_7$	Flavonols	0.3
**3.61**	462.1532	[2-[2-(2-Methoxyphenoxy)ethylamino]-2-oxoethyl] 2-(dimethylamino)-5-nitrobenzoate	$[M-H]^-$	$C_{20}H_{23}N_3O_7$	Alpha amino acids and derivatives	0.3
**7.38**	285.0345	Luteolin	$[M-H]^-$	$C_{15}H_{10}O_6$	Flavones	0.29
**10.39**	241.0759	7,8-dimethylalloxazine (lumichrome)#	$[M-H]^-$	$C_{12}H_{10}N_4O_2$	Flavins	0.28
**12.61**	269.0703	7-O-Methylchrysin	$[M-H]^-$	$C_{15}H_{10}O_5$	Flavonols	0.28
**8.65**	269.0341	3,7,4'-Trihydroxyflavone	$[M-H]^-$	$C_{15}H_{10}O_5$	Flavonols	0.27
**5.85**	191.0808	2-ureidopentanedioic acid	$[M+H]^+$	$C_6H_{10}N_2O_5$	Glutamic acid and derivatives	0.27
**10.59**	301.064	2,5,7-trihydroxy-3-(4-methoxyphenyl)-4H-chromen-4-one	$[M+H]^+$	$C_{16}H_{12}O_6$	Isoflavonoids	0.27
**3.21**	751.2419	Scropolioside A	$[M-H]^-$	$C_{35}H_{44}O_{18}$	Iridoids monoterpenoids	0.26
**10.58**	299.0512	Kaempferol-4'-methyl ether	$[M-H]^-$	$C_{16}H_{12}O_6$	Flavonols	0.25

Average RT (min)	Average m/z (Da)	Tentative ID	Adduct type	Formula	Ontology/Class	Relative %
**8.61**	579.184	CID 131836808	$[M-H]^-$	$C_{31}H_{34}O_{12}$	Flvanone glycoside	0.25
**6.19**	609.1215	Quercetin 3-(2''-P-Coumarylglucoside)	$[M-H]^-$	$C_{30}H_{26}O_{14}$	Flavonoid-glycosides	0.25
**13.39**	867.4362	Cryptostigmin I	$[M-H]^-$	$C_{44}H_{68}O_{17}$	Cardenolides -Triterpenes	0.25
**13**	825.4257	digoxin	$[M+FA-H]^-$	$C_{41}H_{64}O_{14}$	Cardenolides	0.23
**8.37**	276.0597	liriodenine	$[M+H]^+$	$C_{17}H_9NO_3$	Aporphines	0.22
**3.75**	137.019	Protocatechuic aldehyde	$[M-H]^-$	$C_7H_6O_3$	Hydroxybenzaldehydes	0.21
**12**	313.1018	8-(2-hydroxy-1-methoxy-3-methylbut-3-enyl)-7-methoxychromen-2-one	$[M+H]^+$	$C_{16}H_{18}O_5$	Coumarins and derivatives	0.21
**4.55**	135.04	Phenylacetic acid	$[M-H]^-$	$C_8H_8O_2$	Benzene and substituted derivatives	0.2
**6.12**	301.0294	Quercetin #	$[M-H]^-$	$C_{15}H_{10}O_7$	null	0.2
**10.96**	347.1809	Gibberellin A14	$[M-H]^-$	$C_{20}H_{28}O_5$	Diterpenoids	0.2
**7.66**	519.1662	2-Acetonyl-5-methyl-7-methoxy-8-(2-O-trans-cinnamoyl-beta-D-glucopyranosyl)chromone	$[M-H2O-H]^-$	$C_{29}H_{30}O_{10}$	Phenolic glycosides/chromones	0.2
**16.37**	265.135	(1S,8R,9R)-8-hydroxy-4-(propan-2-ylidene)-10-oxatricyclo[7.2.1.01,5]dodecane-8-carboxylic acid#	$[M-H]^-$	$C_{15}H_{22}O_4$	Alpha hydroxy acids and derivatives	0.19

The relative percentage was calculated by dividing the feature intensity by the total intensity of all significant features compared with the blank, using a threshold of $|\log_2 FC| > 1$ and Q-value < 0.05. Feature annotations are reported as tentative LC-MS/MS-based annotations and should not be interpreted as confirmed compound identifications. This supplementary table includes the full phytochemical feature list, including unknown features, duplicated annotations, and features with lower MS/MS matching confidence. Features marked with # indicate selected abundant features prioritised for further SwissADME-based interpretation based on predicted gastrointestinal absorption, blood–brain barrier permeability, and P-glycoprotein substrate status.

Supplementary Table S2. SwissADME-derived molecular ADME properties of selected abundant tentatively annotated JGF features.

ID	Metabolite name	WLOGP	TPSA	GI absorption	BBB permeant	P-gp substrate
1	Baicalein	2.58	90.9	High	No	No
2	3,7,4'-Trihydroxyflavone	2.58	90.9	High	No	No
3	Honokiol	4.22	40.46	High	Yes	No
4	Wogonin	2.88	79.9	High	No	No
5	2'-Hydroxy-4-methoxychalcone	3.19	46.53	High	Yes	No
6	5,7-Dimethoxy-2-[4-[3,4,5-trihydroxy-6-(hydroxymethyl)oxan-2-yl]oxyphenyl]-2,3-dihydrochromen-4-one	0.26	144.14	Low	No	Yes
7	Chrysin	2.87	70.67	High	Yes	No
8	Luteolin-7,3'-di-o-glucoside	-2.77	269.43	Low	No	Yes
9	L-Sinoacutine	1.56	59	High	Yes	Yes
10	(1S,4aS,7S,7aS)-7-hydroxy-7-methyl-1-[(2S,3R,4S,5S,6R)-3,4,5-trihydroxy-6-(hydroxymethyl)oxan-2-yl]oxy-4a,5,6,7a-tetrahydro-1H-cyclopenta[c]pyran-4-carboxylic acid	-2.1	166.14	Low	No	Yes
11	7-O-Methylbaicalein	2.88	79.9	High	No	No
12	Corytenchin / Magnoflorine	2.22	51.16	High	Yes	Yes
13	Berberrubine	2.79	51.8	High	Yes	Yes
14	CID 122229730	1.54	214.06	Low	No	No
15	7,8-dimethylalloxazine (lumichrome)	1.6	92.02	High	No	Yes
16	Harmine hydrochloride	3.84	37.91	High	Yes	Yes
17	2-ureidopentanedioic acid	-1.03	129.72	High	No	No
18	Quercetin	1.99	131.36	High	No	No
19	Chrysin 6-C-glucoside 8-C-arabinoside	-2.11	230.74	Low	No	Yes

20	(1S,8R,9R)-8-hydroxy-4-(propan-2-ylidene)-10-oxatricyclo[7.2.1.01,5]dodecane-8-carboxylic acid	2.12	66.76	High	Yes	Yes

WLOGP indicates predicted lipophilicity, and TPSA indicates topological polar surface area. GI absorption, BBB permeability, and P-gp substrate status were predicted using SwissADME. BBB, blood–brain barrier; GI, gastrointestinal; P-gp, P-glycoprotein; TPSA, topological polar surface area.

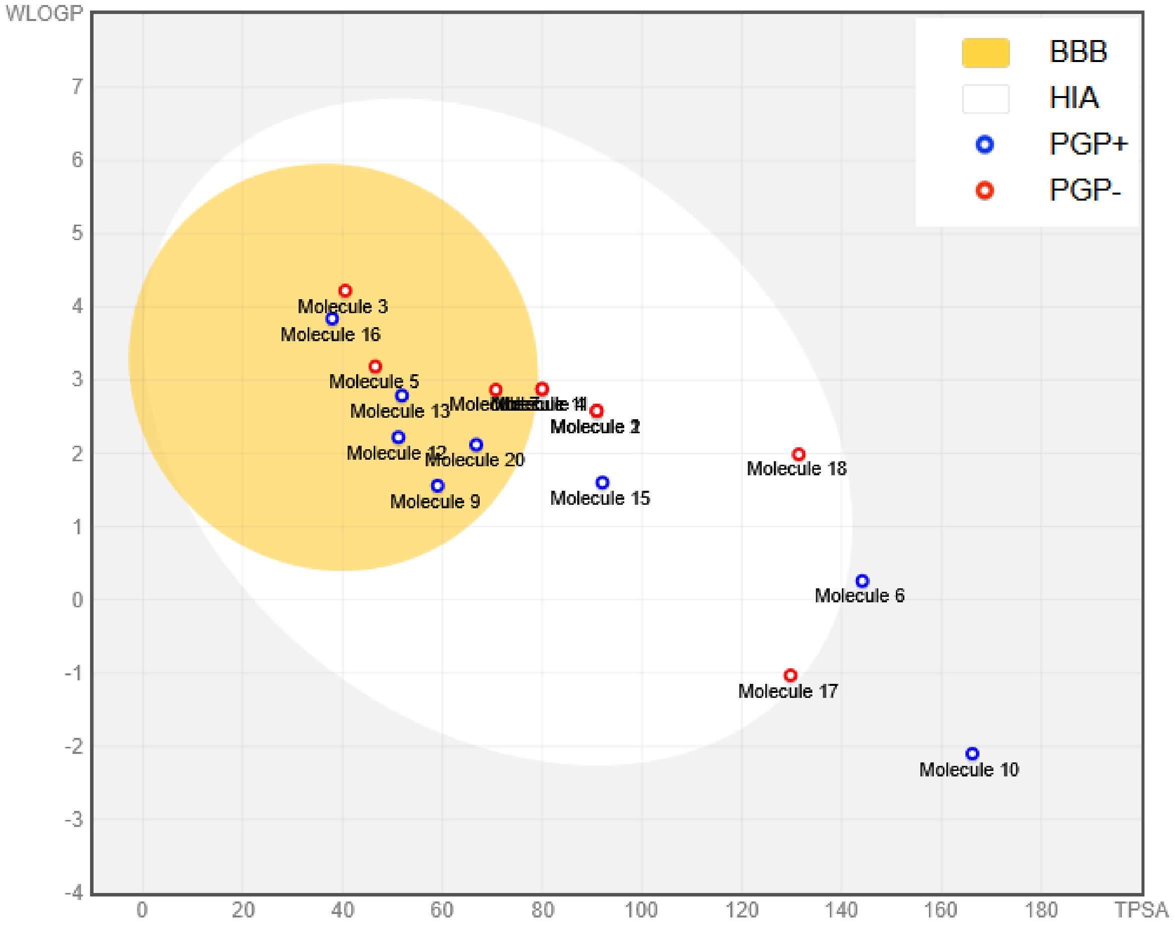

Supplementary Figure S1. BOILED-Egg plot of selected abundant tentatively annotated JGF features showing WLOGP versus TPSA, with predicted blood–brain barrier permeability, gastrointestinal absorption, and P-glycoprotein substrate status. Molecule numbers correspond to those listed in Supplementary Table S2.
